# De la investigación a la acción: estrategias para el manejo de las enfermedades crónicas no transmisibles

**DOI:** 10.7705/biomedica.7354

**Published:** 2023-12-29

**Authors:** Ricardo A. Peña-Silva

**Affiliations:** 1 Facultad de Medicina, Universidad de los Andes, Bogotá, D.C. , Colombia Lown Scholars Program, Department of Global Health and Population, Harvard T. H. Chan School of Public Health, Boston, MA, USA Universidad de los Andes Facultad de Medicina Universidad de los Andes Bogotá, D.C. Colombia

**Keywords:** enfermedades crónicas, cáncer, envejecimiento, fragilidad, inteligencia artificial, obesidad, actividad física

Me gustaría comenzar este editorial con una perspectiva positiva; sin embargo, la gravedad de la situación requiere abordar los desafíos de manera clara con el objetivo de fomentar respuestas efectivas. Las enfermedades crónicas no transmisibles son una amenaza real. En este momento, su control debería ser la máxima prioridad de la salud pública en regiones como Latinoamérica. No obstante, al acercarnos al cuarto de siglo del siglo XXI, observamos que nuestro progreso como sociedad en el control de las enfermedades crónicas no transmisibles aún es insuficiente.

A pesar de contar con datos que demuestran la posibilidad de promover el control de factores de riesgo y adoptar estilos de vida saludables, así como herramientas para diagnósticos oportunos y tratamientos muy efectivos para condiciones comunes, la prevalencia de las enfermedades crónicas no transmisibles sigue en aumento. Este desafío se vuelve aún más complejo al considerar el rápido envejecimiento poblacional en regiones como Latinoamérica, el bajo alfabetismo en salud de la población y la desinformación que circula sobre temas de salud.

Este número especial de la revista *Biomédica* tiene como objetivo concientizar a la población en general, a los profesionales de la salud y a los responsables de las políticas públicas, sobre la importancia de invertir en investigación, diagnóstico y manejo de los factores de riesgo que amenazan la salud de las comunidades. Además, los artículos publicados en este número nos ofrecen perspectivas valiosas sobre elementos que debemos considerar al abordar la gestión de las enfermedades crónicas no transmisibles. En particular, estos artículos analizan:

1. el inadecuado control de algunos factores de riesgo y el poco cumplimiento de los tratamientos;
2. el impacto del envejecimiento y la fragilidad en el control de las enfermedades crónicas no transmisibles y sus complicaciones;
3. la implementación de modelos de diagnóstico y tratamiento oportunos de las enfermedades crónicas no transmisibles en la población, y
4. la utilidad del análisis de los datos en salud para el seguimiento y el control de las enfermedades crónicas no transmisibles.

En primer lugar, en dos artículos se examinan los resultados de la Encuesta Nacional de Salud y Nutrición (ENSIN) 2015. Sus análisis revelan una gran prevalencia de sobrepeso y obesidad en Colombia ^1^, junto con bajos niveles de actividad física, aproximadamente, en la mitad de los adultos ^2^. Tanto el sobrepeso como el sedentarismo aumentan con la edad, pero es preocupante que incluso los valores en jóvenes estén lejos de ser ideales, y alrededor de uno de cada cinco adultos jóvenes tiene sobrepeso.

En una revisión sistemática, se buscó analizar el nivel de observancia a las recomendaciones terapéuticas en Colombia ^3^. Este estudio revela que el cumplimiento de los tratamientos es bajo, en cerca de dos de cada cinco personas, pero las causas no son claras. Es crucial, por lo tanto, trabajar en la alfabetización en salud de las comunidades y combatir la desinformación que se difunde en las redes sociales. El objetivo es empoderar a los pacientes para que adquieran un mejor conocimiento de sus enfermedades, las opciones terapéuticas y la importancia de alcanzar metas terapéuticas adecuadas. La toma de decisiones compartida requiere profesionales de la salud y pacientes que puedan establecer un diálogo respetuoso y basado en la mejor evidencia disponible sobre estrategias para el cuidado de la salud.

También son necesarias políticas e intervenciones que promuevan la salud durante el envejecimiento y en personas con fragilidad física y social. Los estudios en pacientes con complicaciones por cirrosis ^4^, insuficiencia cardíaca ^5^ y diálisis ^6^, demuestran cómo el envejecimiento y la fragilidad asociada a la edad se relacionan con mayores tasas de complicaciones, hospitalizaciones y mortalidad. Un paso necesario en el futuro cercano es comprender los mecanismos y variables que pueden explicar la relación entre la fragilidad, el envejecimiento y las complicaciones por enfermedades crónicas. Entre estas variables, se deben incluir los factores determinantes sociales de la salud y las comorbilidades. El comprender los factores asociados con las complicaciones de las enfermedades crónicas no transmisibles, puede ayudarnos a diseñar estrategias específicas e intervenciones a nivel de sistemas de salud nacionales y locales que han demostrado ser efectivas en su manejo.

El diagnóstico y tratamiento oportuno de los factores de riesgo o de las enfermedades crónicas no transmisibles tienen un gran impacto en su control y en el pronóstico de las personas afectadas por estas condiciones. En este número, tres artículos demuestran la eficacia de las intervenciones tempranas para mejorar el tamizaje de las personas en riesgo de desarrollar cáncer. Los modelos de tamizaje que incluyen pacientes de familiares ya afectados por el cáncer (como es el caso de los síndromes linfoproliferativos) ^8^ o que puedan mejorar la evaluación de los pacientes en alto riesgo (como el sistema OLGA en el cáncer gástrico) ^9^, pueden mejorar el diagnóstico temprano o su seguimiento. El diagnóstico temprano, además, puede ir acompañado de intervenciones terapéuticas tempranas, que pueden estar en manos de diferentes profesionales de la salud. Un ejemplo de esta última estrategia se presenta en el artículo de Pulido y colaboradores, que muestra cómo un grupo de profesionales de enfermería -con entrenamiento especializado- logra buenos resultados terapéuticos con el uso de crioterapia para el manejo de lesiones premalignas del cuello uterino ^10^.

En el abordaje integral de las enfermedades crónicas no transmisibles es importante recordar que una gestión efectiva y la apertura de los datos de salud contribuyen al desarrollo de proyectos de investigación y a optimizar las intervenciones en salud. Hoyos y colaboradores utilizaron datos abiertos de un hospital del sur de Asia para desarrollar un modelo de inteligencia artificial basado en mapas cognitivos difusos, con el fin de mejorar la predicción de qué pacientes podrían desarrollar diabetes ^11^. Los estudios de este tipo subrayan la importancia de contar con una arquitectura de datos en salud que respalde los procesos administrativos, clínicos e investigativos en salud. El compartir estos datos de manera abierta, siguiendo las recomendaciones de los comités de ética y protegiendo la integridad y privacidad de los pacientes, facilita la minería de datos en salud. La analítica de los datos en salud respaldada por la inteligencia artificial tiene numerosas aplicaciones potenciales, como la priorización de las intervenciones de salud personalizadas en momentos críticos del ciclo de vida para predecir, prevenir y desacelerar la aparición de las enfermedades crónicas no transmisibles y sus complicaciones.

Todos formamos parte del ecosistema de datos. La acción de completar adecuada y completamente las historias clínicas, utilizando las definiciones, terminología o códigos CIE-10 apropiados, mejora la calidad de las grandes bases de datos, como SISPRO, que nos ayudan a realizar una gestión e investigación en salud más efectivas.

Este número especial resalta la necesidad de controlar los factores de riesgo para el desarrollo de enfermedades crónicas no transmisibles y destaca la importancia de intervenciones inclusivas que reconozcan la fragilidad de ciertos grupos, especialmente los adultos mayores. El diagnóstico temprano y preciso, junto con las intervenciones terapéuticas oportunas, son cruciales para mejorar los resultados en enfermedades crónicas. Es necesario avanzar en la comprensión de las enfermedades crónicas no transmisibles a nivel molecular. La biología molecular y la biotecnología son esenciales para desarrollar estrategias diagnósticas y terapéuticas más eficaces y personalizadas.

La implementación de estos sistemas respaldados por el análisis de datos y la inteligencia artificial, puede mejorar significativamente la eficiencia de nuestras tareas y la calidad de los resultados. Confiamos en que estos artículos no solo informen y eduquen, sino que, también, inspiren a los profesionales de la salud, a los formuladores de políticas y al público en general a adoptar medidas proactivas en la lucha contra estas enfermedades que afectan a numerosas personas en Latinoamérica y el Caribe.
